# Prevalence of predictive factors for obstructive sleep apnea in university students

**DOI:** 10.5935/1984-0063.20210008

**Published:** 2022

**Authors:** Eduardo Rodrigues dos Santos, Jamilly Henrique Costa da Silva, Anna Myrna Jaguaribe Lima, Luciana Moraes Studart-Pereira

**Affiliations:** 1 Federal University of Pernambuco, Speech-Language-Hearing Department - Recife - Pernambuco - Brazil.; 2 Rural Federal University of Pernambuco, Department of Animal Morphology and Physiology - Recife - Pernambuco - Brazil.

**Keywords:** Apnea, Sleep, Sleep Apnea, Obstructive, Sleep Disorders, Circadian Rhythm

## Abstract

**Objectives:**

To identify predictive factors for obstructive sleep apnea in university students.

**Material and Methods:**

Analytical, observational, cross-sectional study, conducted from August 2018 to February 2019. 144 students of both genders, informed of purposes and procedures, signed consent forms to participate. Procedures included measuring body mass index, abdominal, and neck circumference; inspecting tongue and oropharynx with Mallampati modified classification; answering Berlin questionnaire.

**Results:**

63.9% had normal body mass index; 19.4% were overweight. 59.7% had normal abdominal circumference; 89.6%, normal neck circumference. 65.3% presented Mallampati class I V. 74.3% indicated no obstructive sleep apnea; 25.7% were at high risk. High risk for obstructive sleep apnea associated with body mass index (p<0.001), abdominal (p=0.006), and neck circumference (p<0.001).

**Discussion:**

Anthropometries were mostly normal, despite the high prevalence of changed Mallampati classification. Positively correlated predictive factors, also associated with high risk for obstructive sleep apnea, reinforce the need for such preventive measures in youth.

## INTRODUCTION

Sleep disorders in youth have been often reported in the literature, as well as short sleep duration, which has been associated with low academic performance. Research shows that students have irregular sleep patterns due to excessive obligations, such as classes, night shift jobs, and social events^1^. Diminishing or missing one’s rest time can trigger various clinical conditions, with countless consequences, for instance, on their wakefulness, quality of life, and the onset of various diseases^2^.

Obstructive sleep apnea (OSA) is a sleep disorder characterized by paused (apnea) or shallow (hypopnea) flow of air through the upper respiratory tract^3^. The prevalence of OSA in mouth breathers and obese people highlights the need to identify predictive factors^4^. Excessive adipose tissue in the obese people’s upper respiratory tract narrows it, making it more prone to collapse during sleep and hinder the airflow, which causes apnea and hypopnea^5^.

Hence, the predictive factors for OSA among young people must be identified^6^. Maurício et al. (2018)^7^ point out both modifiable and hereditary risk factors associated with the cardiovascular system – e.g., overweight/obesity – which may be related to complications resulting from OSA. In such a context, the younger population’s living habits must also be considered.

This disease may be triggered by factors related to the body and oromyofacial structures, as well as the person’s lifestyle and habits. Hence, identifying sleep respiratory disorders as soon as possible and making people aware of them allow for early prevention and health promotion with clinical and/or surgical intervention. Thus, this study aimed to identify the prevalence and correlation of predictive factors for OSA in university students.

## MATERIAL AND METHODS

This analytical, observational, cross-sectional study was approved by the Research Ethics Committee of the Federal University of Pernambuco (UFPE) (No. 2.891.458). The participants were informed of the purpose of the study and collection procedures. Having agreed to participate in the research, they signed the informed consent form (ICF).

The collection took place from August 2018 to February 2019, in a speech-language-hearing clinic at UFPE. The sample comprised 144 dentistry and speech-language-hearing students, mean age 33.79 years. The inclusion criteria established for this investigation were: actively enrolled university students of both sexes. The exclusion criteria were: students on a leave of absence from college, away in an exchange program, with disabilities, neurological or psychiatric problems that hindered their communication.

The students had their weight measured in the anthropometric assessment, stepping barefoot up onto a digital scale with a maximum capacity of 180kg and gradation/ precision of 100g. Their height was measured with a millimeter measuring tape attached to the wall, beside which the students stood upright and barefoot, their heels, back, and head touching the wall and facing forward. A room in each department was set apart for this procedure, which lasted approximately 25 minutes.

The weight and height measures were used to calculate their BMI [weight (kg)/height(m)²]. The neck circumference (NC) was measured right below the laryngeal prominence, in the neutral head position. NC measuring more than 38mm for women and 40mm for men were considered predictive values for OSA.

The abdominal circumference (AC) was measured in a standing position, with the abdomen relaxed. The measuring tape was placed horizontally in the midpoint between the lower rim of the last rib and the iliac crest. The cutoff scores pointing to increased risk were 80cm for women and 94cm for men.

The tongue and oropharynx were inspected with Mallampati modified classification, by an assessor familiarized with the oral and pharyngeal cavity structures. The participants were seated with their heads upright, and the assessor asked them to swallow and open their mouth and keep their tongue relaxed.

Two photographs of each participant’s oral cavity were taken. The images were selected for analysis based on the best luminosity and position of the tongue. The chosen images were organized in slide presentations and separately sent to three assessors (a speech-language-hearing therapist certified in sleep medicine, an anesthesiologist, and an otorhinolaryngologist), along with an answer sheet. The professionals were requested to observe the relationship between the tongue and the oral cavity and classify it according to Mallampati modified score as class I, II, III, or IV^8^. After analyzing the images and filling out the answer sheet, it was returned via e-mail, blinded, and forwarded for statistical analysis. Whenever the three classifications did not coincide, two decision criteria were used: the partial, when two assessors agreed; and the middle, when the three assessors disagreed – in which case the intermediate result was considered.

Afterward, the participants answered the Berlin questionnaire – a screening tool for OSA. It is a simple and validated method that identifies high risk for OSA with questions divided into three categories. Category 1 refers to snoring; category 2 approaches day sleepiness; category 3 is on the history of systemic arterial hypertension (SAH) and body mass index (BMI). The categories were scored separately, adding one point to every affirmative answer. Categories 1 and 2 were considered positive when the total score was 2 or more, and category 3, when the person had SAH or obesity (BMI of 30kg/m^2^ or higher). Those with two or more positive categories were classified at high risk for OSA.

The weighted kappa index and confidence interval were obtained to verify the degree of coincidence between each pair of assessors, as well as for the three pairs of assessors. The MEDCALC statistical program was used, version 14.8.1, and the intervals were obtained with 95% reliability.

The other data were presented in absolute and percentage frequencies for the categorical variables, and statistics (mean, standard deviation, and median) for age. The Pearson chi-square test was used to assess the association of each dependent variable with the independent ones. When the conditions to use the chi- square test were not met, Fisher’s exact test was used.

The margin of error used in decisions in the statistical tests was 5% and the intervals for the odds ratio were obtained with 95% confidence. The IBM SPSS, version 23, was used to enter the statistical data and develop the results of the degree assessments of the statistical calculations.

## RESULTS

Most of this sample were females (70.1%); 66.7% were dentistry students, and 33.3% were speech-language-hearing students. A greater percentage studied in the morning (41.7%), while 33.3% studied full-time, and 25.0% studied at night.

As seen in Table 1, most of the students (63.9%) had normal BMI, followed by 19.4% overweight. The obese and the underweight corresponded, respectively, to 10.4% and 6.3%. There was a prevalence of 40.3% of students with high AC, whereas the others had a normal measure. The NC was high in 10.4% of them, and normal in the others.

**Table 1.** Prevalence of predictive factors: body mass index, abdominal circumference, neck circumference, and Mallampati classification, Recife, 2019.

Variable	N	%
Total	144	100.0
**BMI**		
<Underweight	9	6.3
Normal weight	92	63.9
Overweight	28	19.4
Obesity	15	10.4
**Abdominal circumference**		
High	58	40.3
Normal	86	59.7
**Neck circumference**		
High	15	10.4
Normal	129	89.6
**Mallampati**		
I	16	11.1
II	20	13.9
III	14	9.7
IV	94	65.3
**Berlin Questionnaire**		
High risk for OSA	37	25.7
Not indicative of OSA	107	74.3

Notes: BMI = Body mass index; OSA = Obstructive sleep apnea.

Table 1 approaches the Berlin questionnaire as well, in which most of the students’ results (74.3%) were not indicative of OSA, while the percentage of those at high risk for OSA was 25.7%.

Also, most of the students (65.3%) presented Mallampati modified class IV; classes I, II, and III had the percentages of 11.1%, 13.9%, and 9.7%, respectively. The results of each assessor’s Mallampati class assessment, as well as the interassessor agreement, are seen in Table 2. Most of the assessors classified the students in class IV (percentages ranging from 61.8% to 68.1%). The percentage of those in class I ranged from 4.2% to 13.2%; in class II, from 6.9% to 15.3%; and in class III, from 11.1% to 20.8%. The weighted kappa index was higher between assessors 1 and 2 (0.92), indicating an excellent agreement; between the other two pairs, it was 0.69 and 0.72 (good agreement). The kappa index for the three pairs of assessors was 0.78 (good agreement).

**Table 2.** Results of Mallampati class assessment per assessor, Recife, 2019.

	Assessor				
Mallampati class	1 n (%)	2 n (%)	3 n (%)	Pairs of assessors	Weighted kappa 95% CI
				0.92 (0.88 to 0.96)	
**I**	15 (10.4)	19 (13.2)	6 (4.2)	1 x 2	0.92 (0.88 to 0.96)
**II**	22 (15.3)	16 (11.1)	10 (6.9)	1 x 3	0.69 (0.62 to 0.75)
**III**	18 (12.5)	16 (11.1)	30 (20.8)	2 x 3	0.72 (0.65 to 0.79)
**IV**	89 (61.8)	93 (64.6)	98 (68.1)		
**Total**	**144**	**144**	**144**	**3 pairs of assessors**	0.78 (0.72 to 0.83)
	**(100.0)**	**(100.0)**	**(100.0)**		

Notes: CI = Confidence interval; Kappa index: <0.20 poor; 0.21-0.40 weak; 0.41-0.60 moderate; 0.61-0.80 good; 0.81-0.99 excellent; 1.00 perfect.

In Table 3, a weak correlation can be verified between age and BMI (r=0.28); age and AC (r=0.27); age and NC (r=0.18); Mallampati and BMI (r=0.24); and between Mallampati and AC (r=0.17). Also, there were moderate correlations between BMI and NC (r=0.59); and between AC and NC (r=0.64). And lastly, there was a strong correlation between AC and BMI (r=0.82).

**Table 3.** Spearman’s correlation between age, body mass index, abdominal circumference, neck circumference, and Mallampati, Recife, 2019.

Variable	Age	BMI	AC	NC
Age				
BMI	0.28 (0.001)*			
AC	0.27 (0.001)*	0.82 (<0.001)*		
NC	0.18 (0.028)*	0.59 (<0.001)*	0.64 (<0.001)*	
Mallampati	0.07 (0.381)	0.24 (0.004)*	0.17 (0.043)*	0.12 (0.168)

BMI = Body mass index; AC = Abdominal circumference; NC = Neck circumference; Spearman correlation; (*) Statistically different from zero.

Table 4 shows the risk for OSA according to BMI, AC, NC, and Mallampati classification. It was verified that 86.7% of those whose BMI characterized them as obese were at high risk for OSA. Although 62.1% of the people with high AC had a normal result in the Berlin questionnaire, 37.9% were at high risk for OSA. As for those with high NC, 66.7% were at high risk for OSA.

**Table 4.** Assessment of risk for obstructive sleep apnea, according to body mass index, abdominal and neck circumference, and Mallampati classification.

Berlin questionnaire					
Variable	High risk for OSA		Not indicative of OSA		*p*-value N %
	N	%	N	%	
**BMI**					*p*^(2)^ < 0.001*
< Underweight	3	33.3	6	66.7	
Normal weight	16	17.4	76	82.6	
Overweight	5	17.9	23	82.1	
Obesity	13	86.7	2	13.3	
**Abdominal circumference**					*p*^(1)^ = 0.006*
High	22	37.9	36	62.1	
Normal	15	17.4	71	82.6	
**Neck circumference**					*p*^(2)^ < 0.001*
High	10	66.7	5	33.3	
Normal	27	20.9	102	79.1	
**Mallampati**					*p*^(2)^ = 0.611
I	2	12.5	14	87.5	
II	6	30.0	14	70.0	
III	4	28.6	10	71.4	
IV	25	26.6	69	73.4	

Notes: BMI = Body mass index; OSA = Obstructive sleep apnea; (*) Significant association at 5%; (1) With the Pearson chi-squared test; (2) With Fisher’s exact test.

There was a statistical significance in the investigated variables.

Table 4 also presents the percentage of people at high risk for OSA in Mallampati class I (12.5%), class II (30%), class III (28.6%), and class IV (26.6%).

## DISCUSSION

Although most of the university students had normal OSA-related anthropometric values, there was a greater prevalence of changes in Mallampati class IV. Moreover, weak, moderate, and strong correlations were found in the anthropometric variables, both with one another and with the Mallampati index.

Sleep deprivation is today a public health issue associated with obesity, morbidity, and mortality. Studies show that less than six hours of sleep each night is related to increased BMI and obesity^9,10^. Restricted sleep is related to increased appetite and consumption of more caloric foods, resulting in weight gain. A study by Souza et al. (2017)^11^ analyzed students and concluded that the participants were at great risk of developing overweight and obesity because of both sedentarism and poor eating habits. It also concluded that such a condition could result from sleep deprivation due to their stressful routines. Although the population studied in this research did not have alarming body weight indexes, there was an association between high BMI and high risk for OSA, according to the Berlin questionnaire. In this research, there was a prevalence of 19.4% of overweight and 10.4% of obesity. Although these are not high results, they indicate that one third of this sample was above their ideal weight, possibly due to inadequate eating habits, lifestyle, and sleep deprivation, as shown in Brazilian research^7,12^.

The World Health Organization points out that obesity is one of the greatest worldwide public health problems. The projection is that, by 2025, approximately 2.3 billion adults will be overweight, and over 700 million, obese^13^. These projections may relate to a likely growth in sleep disorders. Excessive weight is an important predictor for OSA, due to changed anthropometric measures and soft tissues in the oral cavity, tongue, and oropharynx.

Predictive factors for OSA are described in research by Pinto et al. (2011)^14^ and Zimberg et al. (2017)^15^. They show that, although some factors are more efficient than others, investigating them together ensures greater reliability in prior identification.

This study identified many changes in Mallampati classification, despite the few changes in the other factors. This reinforces the importance of analyzing the factors together and in a context. Findings from Dias et al. (2015)^16^ and Araújo-Melo et al. (2016)^17^ showed that Mallampati classification is a predictive factor that presents considerable changes even in people without the disease.

The tendency of orofacial myofunctional changes in individuals with OSA has been demonstrated in another study, revealing that the greater the degree of structural impairment, the greater the severity of the disease^18^.

In this study, moderate to strong correlations were observed between the BMI and the AC and NC, and between the NC and AC. The anthropometric and orofacial measures are useful in identifying individuals at risk of OSA^19^. Amra et al. (2019)^20^ consider that the anthropometric measures and Mallampati index are important to predict moderate and severe OSA.

These results call attention to predictive factors for OSA and the need for contextualized assessment. Even without a definite diagnosis, it is necessary to observe the predictive factors among students. Besides encouraging them to undergo examinations (such as polysomnography), they help clinical professionals identify onsetting diseases.

Some limitations were perceived in the study, such as the students’ availability to participate only in the brief spare time they had. To minimize it, the collection was rescheduled to match the students’ timetable.

It is concluded that most of the population studied had anthropometric data within normal standards. However, a high prevalence of changed cases in Mallampati classification was verified. The positive correlation between the predictive factors and their association with high risk for OSA point to the need for preventive measures of OSA also in the young population.
